# Adaptive Evolution of *Leptin* in Heterothermic Bats

**DOI:** 10.1371/journal.pone.0027189

**Published:** 2011-11-16

**Authors:** Lihong Yuan, Xudong Zhao, Benfu Lin, Stephen J. Rossiter, Lingjiang He, Xueguo Zuo, Guimei He, Gareth Jones, Fritz Geiser, Shuyi Zhang

**Affiliations:** 1 South China Institute of Endangered Animals, Guangzhou, China; 2 Key Laboratory of Marine Bio-resources Sustainable Utilization, Key Laboratory of Applied Marine Biology of Guangdong Province, South China Sea Institute of Oceanology, Chinese Academy of Sciences, Guangzhou, China; 3 Institute of Zoology, Chinese Academy of Sciences, Beijing, China; 4 School of Biological and Chemical Sciences, Queen Mary University of London, London, United Kingdom; 5 Institute of Molecular Ecology and Evolution, Institutes for Advanced Interdisciplinary Research, East China Normal University, Shanghai, China; 6 School of Biological Sciences, University of Bristol, Bristol, United Kingdom; 7 Center for Behavioural and Physiological Ecology, Zoology, University of New England, Armidale, Australia; University of Western Ontario, Canada

## Abstract

Heterothermy (hibernation and daily torpor) is a key strategy that animals use to survive in harsh conditions and is widely employed by bats, which are found in diverse habitats and climates. Bats comprise more than 20% of all mammals and although heterothermy occurs in divergent lineages of bats, suggesting it might be an ancestral condition, its evolutionary history is complicated by complex phylogeographic patterns. Here, we use *Leptin*, which regulates lipid metabolism and is crucial for thermogenesis of hibernators, as molecular marker and combine physiological, molecular and biochemical analyses to explore the possible evolutionary history of heterothermy in bat. The two tropical fruit bats examined here were homeothermic; in contrast, the two tropical insectivorous bats were clearly heterothermic. Molecular evolutionary analyses of the *Leptin* gene revealed positive selection in the ancestors of all bats, which was maintained or further enhanced the lineages comprising mostly heterothermic species. In contrast, we found evidence of relaxed selection in homeothermic species. Biochemical assays of bat Leptin on the activity on adipocyte degradation revealed that Leptin in heterothermic bats was more lipolytic than in homeothermic bats. This shows that evolutionary sequence changes in this protein are indeed functional and support the interpretation of our physiological results and the molecular evolutionary analyses. Our combined data strongly support the hypothesis that heterothermy is the ancestral state of bats and that this involved adaptive changes in *Leptin*. Subsequent loss of heterothermy in some tropical lineages of bats likely was associated with range and dietary shifts.

## Introduction

The ability of heterothermic mammals to regulate or vary their body temperature (T_b_) in response to changes in ambient temperature (T_a_) and energy requirements is considered key to their evolutionary success, allowing them to survive in adverse climates and during periods when food is scarce [1], [2]. Heterothermy contrasts with homeothermy, characterized by a more or less constant and high T_b_ largely irrespective of T_a_ [3]. Heterothermy in mammals has been categorized as either daily torpor or prolonged multi-day torpor (hibernation), which the duration of torpor bouts last for less than 24 h and T_b_ maintained around 15°C–25°C in the former whereas between 100 h to 1000 h and T_b_ of 0–10°C in the latter [4]. Comparative data show that heterothermy is employed for energy conservation by members of at least ten orders of mammals, including monotremes, four marsupial orders, carnivores, rodents, eulipotyphlans (insectivores), bats and primates [5], [6], [7], [8], [9].

Bats (order Chiroptera) number over 1100 species and are among the most geographically widespread of mammals [10]. Most bat families contain species that can enter daily torpor, whereas hibernation is confirmed in at least seven families [1], [11], [12]. Hibernating species of bats (some members of the superfamilies Emballonuroidea, Vespertilionoidea and Rhinolophoidea) are distributed in cool temperate or subtropical regions, whereas homeothermic bats are mainly tropical or subtropical [13]. Despite these differences in distribution and thermoregulatory patterns, little is known about the evolutionary history of heterothermy in bats. This is complicated further because phylogenetic reconstructions indicate that hibernation occurs in several branches across the mammalian tree [14]. However, currently there is no obvious molecular marker that can be reliably associated with hibernation.

All heterothermic mammals, including hibernating bats require efficient use of adipose tissue because they rely on fat as the primary energy source during periods of torpor, which in turn permits survival of exceedingly low T_a_ and scarce food [7]. Hibernators must have an accurate and efficient way to control lipolysis, not only to control the time point of metabolizing fat for heat production during periodic rewarming, but also to extend the energy supply throughout long winters. The hormone Leptin has been shown to be involved in lipid metabolism and the provision of energy via lipolysis [15]. Metabolism and food intake in hibernating species appears to be regulated by periodic changes in the level of serum Leptin and other related molecules [16], [17], [18], [19], [20]. It was recently suggested that exposure to cold environments has driven the evolution of *Leptin* by selected site substitution in one group of lagomorphs [21]. Such studies implicate Leptin in the control of thermogenesis, crucial for successful hibernation, and therefore the evolution of *Leptin* in hibernators is also likely to be driven by natural selection.

To tease apart the molecular evolution of bat *Leptin*, and explore the possible evolutionary history of heterothermy (hibernation and torpor) in bats, we employed three approaches: (i) we compared thermoregulation in four tropical/subtropical species, two Old World fruit bats and two insectivorous bats, thought to differ in their capacity for expression of heterothermy; (ii) we reconstructed the ancestral states and undertook evolutionary and phylogenetic analyses of the *Leptin* gene in a range of heterothermic and homeothermic bat lineages (see Table S1); and (iii) we expressed two recombinant proteins of bat Leptin with glutathione-S-transferase (GST) tags in *E. coli* cells and used them for bio-activity assays *in vitro* to compare the effect of Leptin on adipocyte lipolysis between heterothermic and homeothermic bats. As lipolysis is a process that provides chemical energy by adipocyte degradation, the lipolytic activity of Leptin is reflected by adipocyte viability, which can be assessed by the reduction of the number of adipocytes, or via quantifying release of Lactase dehydrogenase (LDH) into the media.

## Materials and Methods

### Ethics statement

The physiological experiment and sampling were approved by the Administrative Panel on Laboratory Animal Care of Institute of Zoology, Chinese Academy of Sciences, and accord with the guidelines for the National Care and Use of Animals approved by the National Animal Research Authority (approval ID 20080209).

### Study of thermoregulatory ability

Four similar-sized species of bats (2 pteropodids: *Cynopterus sphinx* and *Eonycteris spelaea*, 1 hipposiderid: *Hipposideros armiger* and 1 vespertilionid: *Scotophilus heathii*) from subtropical and tropical zones were chosen for the thermoregulatory study. All bats were collected in the southeast of China, and were housed individually in separate cages and supplied with food/water and a 12:12 light: dark cycle (lights on from 06:00 to 18:00 h). A telemetry T_b_ transmitter (DSI, USA) was surgically implanted and used to record the T_b_ responsing to T_a_ which change from 25°C to 5°C. One-way analysis of variance (ANOVA) was used for comparison of mean T_b_ of each individual of the four species at the three T_a_s measured (see Text S1).

### Molecular phylogenetic analysis

To assess the evolutionary history of *Leptin* in heterothermic bats, we carried out analyses of molecular evolution in a range of heterothermic and homeothermic bat lineages.

#### Reconstruction of ancestral characters

Based on the composite species tree [22], [23], we reconstructed the ancestral states with binary data (heterothermy vs. homeothermy) and multistate data (hibernation, torpor, homeothermy), using the parsimony and likelihood methods in MESQUITE2.1 [24], to infer the lineages which are potentially related with the evolution of heterothermy.

#### Sequencing and database searches

The Leptin complete CDS or exon 3 were cloned and sequenced from 19 bat species, including six species of the family Pteropodidae: *Eidolon helvum*, *Eonycteris spelaea*, *Rousettus leschenaultii*, *Dobsonia viridis*, *Cynopterus sphinx*, *Pteropus giganteus*; and 13 species from other families: *Taphozous melanopogon* (Emballonuridae), *Miniopterus fuliginosus* (Miniopteridae), *Scotophilus heathii*, *Myotis ricketti* (Vespertilionidae), *Chaerephon plicatus*, *Tadarida teniotis* (Molossidae), *Pteronotus parnellii* (Mormoopidae), *Artibeus gnomus*, *Anoura geoffroyi*, *Carollia brevicauda* (Phyllostomidae), *Hipposideros armiger* (Hipposideridae), *Rhinolophus ferrumequinum* (Rhinolophidae), *Rhinopoma microphyllum* (Rhinopomatidae) (see Text S1 and Table S2). Moreover, the complete *Leptin* sequences of a further 27 mammal species were obtained from the NCBI Nucleotide collection (nr) and Whole-genome shotgun (wgs) databases [25], including six primates: *Homo sapiens*, *Pan troglodytes*, *Pongo abelii*, *Macaca mulatta*, *Otolemur garnettii* and *Microcebus murinus*; two rodents: *Rattus norvegicus* and *Mus musculus*; eight lagomorphs: *Lepus oiostolus*, *Oryctolagus cuniculus*, *Ochotona dauurica bedfordi*, *Ochotona annectens*, *Ochtona nubrica*, *Ochotona curzoniae*, *Ochotona cansus cansus* and *Ochotona princeps*; three artiodactyls: *Sus scrofa*, *Bos taurus* and *Capra hircus*; one perissodactyl: *Equus caballus*; three carnivores: *Felis catus*, *Ursus thibetanus japonicus* and, *Canis lupus familiaris*; one bat: *Myotis lucifugus*; one proboscidean: *Loxodonta africana*; one marsupial: *Monodelphis domestica*, and one monotreme: *Ornithorhynchus anatinus*.

#### Evolutionary analyses

Nucleotide sequences were aligned by using ClustalX1.81 [26], and translated in MEGA3.1 [27]. The best-fit model of molecular evolution were determined by Modeltest 3.7 [28] and the phylogenetic trees of *Leptin* were constructed by MrBayes 3.1.1 [29].

To identify the variable selective pressures in the *Leptin*, both across the tree and along specific lineages, we carried out the CODEML program analyses in the PAML package: a free-ratio model, site-specific models, a branch-specific model (two-ratio), and a branch-site model (Model A) [30] (Text S1). We also included published sequence data from the lagomorph group of pikas, in which molecular evolution of *Leptin* has previously been shown to be linked to cold environmental stresses [21].

To resolve further the distribution in selective pressures on specific functional domains of *Leptin* among different clades, we presented Nei and Gojobori [31] substitution rates (*d*_N_ and *d*_S_ per site and the *d*_N_/*d*_S_ ratio) as sliding windows (window = 45 codons, step = 3 codons) for exon 3 using the software SWAAP 1.0.2 [32]. This was repeated for three groups of taxa: 21 placental mammals excluding *U. thibetanus japonicus*and, *M. murinus* and *M. musculus*, 14 heterothermic bats, 6 homeothermic bats.

To test whether the amino acid replacements in the Leptin protein of heterothermic bats are likely to have an impact on protein function, we undertook a multivariate based analysis of protein physicochemical properties using the program MAPP [33]. For this analysis, amino acid sequences of *Leptin* exon 3 orthologues of 27 homeothermic placental mammals and 14 heterothermic bats were aligned, respectively, and weighted by known phylogenetic relationships. From the variation in the dataset, the physicochemical constraint of each site was estimated based on hydropathy, polarity, charge, volume and free energy, and to predict the impact of all possible replacements seen in hibernating bats (MAPP score).

#### Bio-activity assay of Leptin on thermoregulation

Leptin can directly act on adipocyte lipolysis *in vitro* and *in vivo* [34], [35]. To validate differences in activity of Leptin lipolysis between heterothermic and homeothermic bats, the recombinant Leptin proteins of a hibernating species (*M. fuliginosus*) and a homeothermic species (*R. leschenaultii*) were fused with glutathione-S-transferase (GST) tags and expressed in *E. coli* cells. Then, the lipolytic activity of GST-Leptin was measured by the inhibitory effect on cell viability and cytotoxicity on adipocytes.

*Leptin* coding sequences of the two bats, excluding the first 21 amino acids signature domain were amplified by PCR (see Text S1). 3T3-L1 mouse embryo fibroblasts (adipose-like cells, ATCC) were cultured according to the methods described by Hemati et al [36], and used for Leptin activity assay. The effect of Leptin on adipoctytes is reflected by cell viability. One assessment of cell viability is to assess the number of viable cells based on the mitochondrial-dependent reduction of 3-(4,5-dimethylthiazol-2-yl)-2,5-diphenyl tetrazolium bromide (MTT) method. Another is to measure the Leptin cytotoxicity by Lactate dehydrogenase (LDH) assay using the CytoTox-ONETM assay kit (Promega), as the presence of LDH in media relative to its activity in cells is used as criterion of direct cytotoxicity. In our study, 3T3-L1 cells were incubated with bat GST-Leptin (10^−6^ M) at 24 h or 48 h. Experiments were repeated three times with four to six replicates for each treatment. Details of procedures followed are described by Ambati et al. [34], and a *t*-test was used to compare the means of the two bat species.

## Results

### Thermoregulatory study

**Figure 1:**
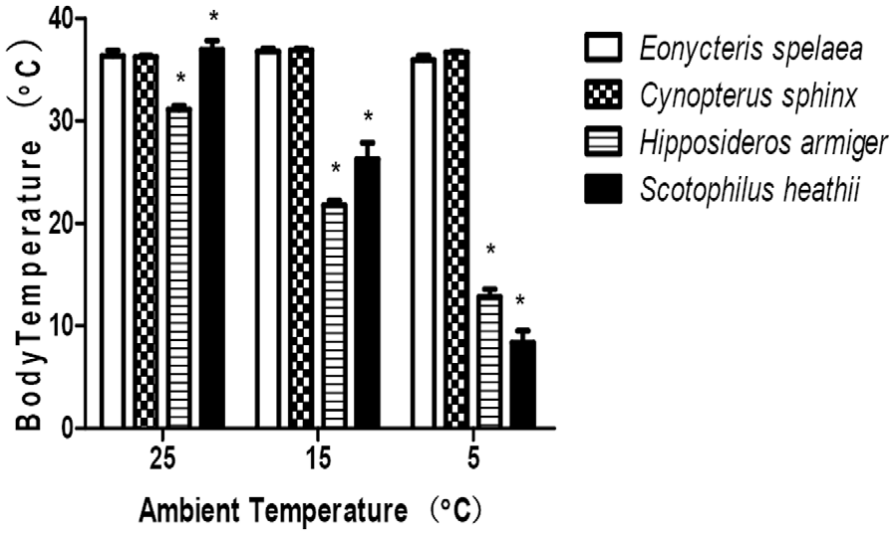
Comparison of body temperature of four bats as a function of ambient temperature. *Eonycteris spelaea* (N = 6), *Cynopterus sphinx* (N = 6), *Hipposideros armiger *(N = 6) and *Scotophilus heathii* (N = 6). The SE is indicated by the error bars. *P<0.0001.

*Cynopterus sphinx* and *Eonycteris spelaea* (Pteropodidae) did not enter torpor or hibernation at all T_a_s measured (Figure 1). The T_b_ of individuals was little affected by T_a_ and mean T_b_ was 36.4±0.2°C for *C. sphinx* (N = 6, n = 36) and 36.8±0.5°C for *E. spelaea* (N = 6, n = 36). In contrast, *Scotophilus heathii* and *Hipposideros armiger* entered torpor, at T_a_ 15°C and 5°C. At T_a_ 15°C and 5°C, the average T_b_ were 26.29±1.58°C and 8.34±1.16°C for *S. heathii*, respectively (F_(2,15)_  = 133.58; P<0.0001; One Way ANOVA); at the same Ta condition, Tb of *H. armiger* were 21.78±0.46°C and 12.81±0.77°C (F_(2,15)_  = 261.6; P<0.0001; One Way ANOVA), respectively. When the T_a_ decreased, *C. sphinx*and *E. spelaea* remained active and ate more food than at T_a_ 25°C; no sign of discomfort was observed. In contrast, torpor in *S. heathii* and *H. armiger* at T_a_ 15°C was observed on both measurement days, but bats showed full or partial rewarming daily; at T_a_ 5°C both species hibernated without rewarming over the two days.

### Evolutionary and selective pressure analysis

**Figure 2:**
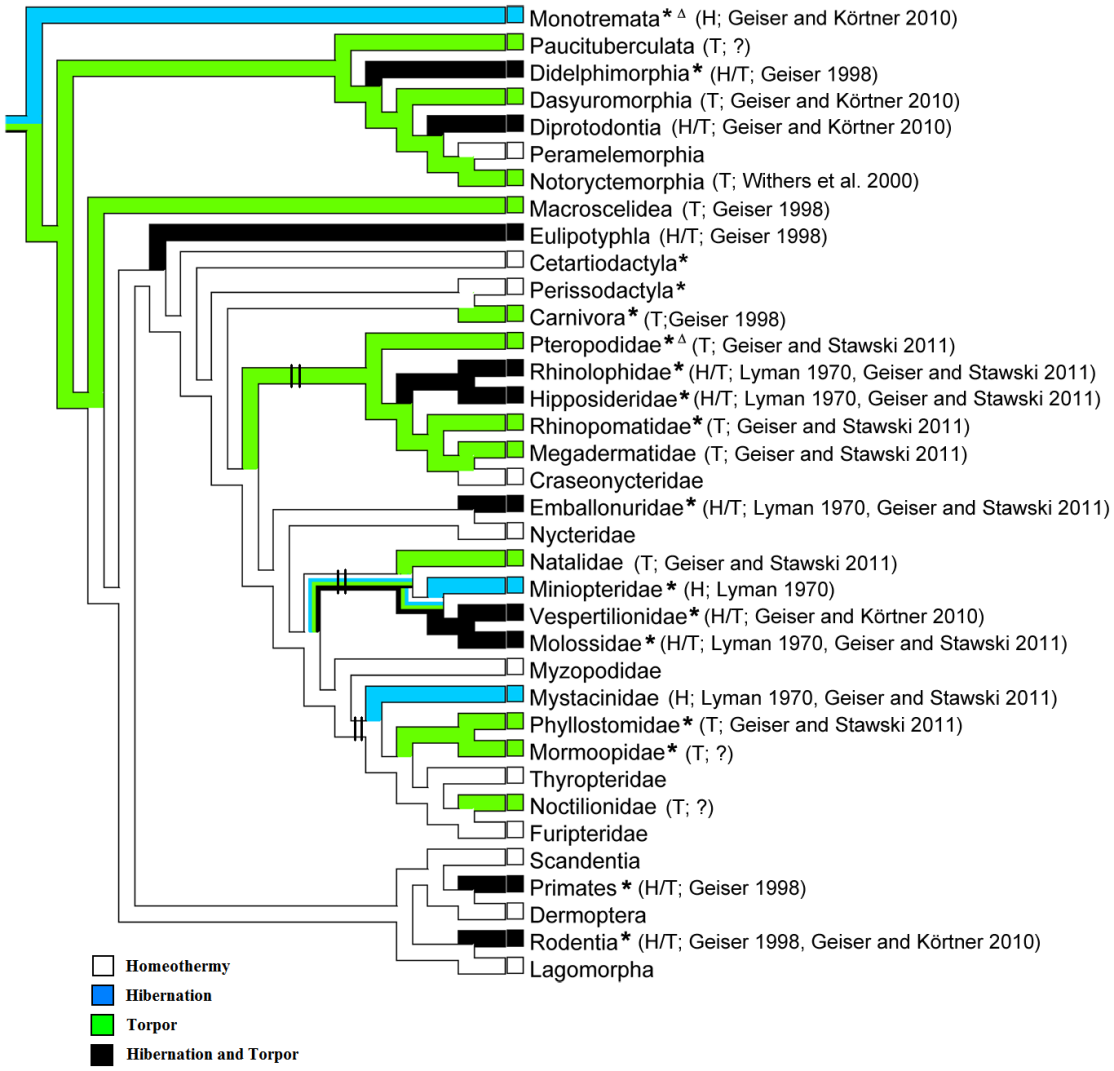
Reconstruction of ancestral states. Multistate data of homeothermic and heterothermic (hibernation and/or torpor) states are mapped onto the composite species tree (Teeling et al. 2005 and Nishihara, Hasegawa, and Okada 2006). Branches show the species of family are homeothermic (white), hibernators (blue), torpor (green), or hibernators and torpor (black). *: some species of this family were test in this study; Δ: species studied here are homeothermic. ?: some species of this family are likely heterothermic. Vertical bars on branches represent the lineages which are potentially related with the evolution of bat heterothermy.

Tracing character history of ancestors indicated that bat heterothermy mainly evolved in three lineages (Figure 2 and Figure S1). We hypothesized that, if *Leptin* is the key of bat heterothermic evolution, natural selected pressure on *Leptin* may be detected in these lineages. To test this hypothesis, we cloned the complete *Leptin* CDS from five bat species and *Leptin* exon 3 from 14 bat species (accession numbers GU230829- GU230833 and GU230835-GU230848) are listed in Table S3.

The *Leptin* gene tree based on the complete CDS, constructed by MrBayes 3.1.1 [29] according to the best-fit model (K80+G) determined by Modeltest 3.7 [28] is shown in Figure S2. The tree topology was consistent with that of previous multigene phylogenies of mammals [23], and this topology was then constrained in subsequent estimates of substitution rates. The free-ratio model showed that the ω ratio was considerably higher in the ancestor of the strepsirrhines (non-tarsier prosimians) (ω = 999.000, N*dN = 4.3, S*dS = 0), the hominid lineage (ω = 2.048, N*dN = 6.1, S*dS = 0), the chiropteran ancestor (ω = 616.224, N*dN = 5, S*dS = 0), the yangochiropteran lineage (ω = 1.225, N*dN = 13.1, S*dS = 3.5) and the rhinolophoid lineage (ω = 1.364, N*dN = 70.4, S*dS = 17.0) than in other lineages, and the ω ratio in the pika lineage was 0.544 (Figure S2). The LRT statistic of the free-ratio model vs. M0 was much greater than critical values from a χ^2^ distribution with 2*Δℓ* = 129.86 (P<0.001 and d.f. = 60) (Table S4). Branch-specific analysis confirmed the selective pressure on these specific lineages with P<0.001 (Table S4). Site-specific analyses indicated that the selective pressure was highly variable among sites, as M0 was rejected by a big margin compared with M3 with 2*Δℓ* = 180.28 (P<0.001 and d.f. = 4), and 15 natural selected sites were identified (Table S4). In addition, model M2a and M8 suggested that 0.78% and 1.23% of sites were under positive selection with ω>1, although the LRT of M8/M8a and M2a/M1a did not achieve significance (Table S4).

**Figure 3:**
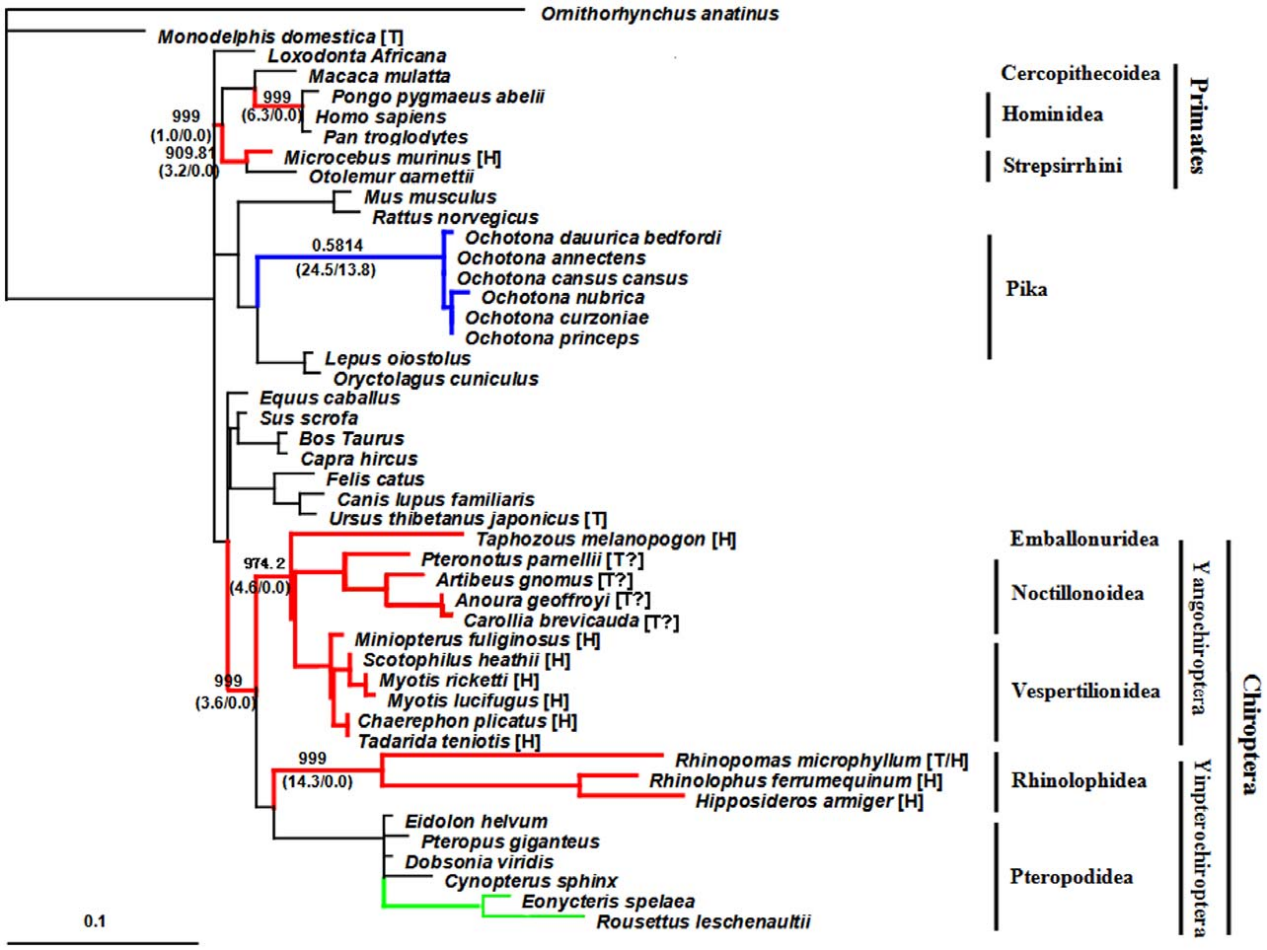
The phylogenetic gene tree of *Leptin*. The gene tree was based on nucleotide sequences of *Leptin* exon 3 and used for the codon maximum likelihood analysis. The ω ratios and numbers of non-synonymous substitution (N*dN) and synonymous substitution (S*dS) of some specific lineages were shown in bold and in bracket. The scale bar of “0.1” means 0.1 nucleotide substitution per site. [T], torpor; [H], hibernator. Lineages with ω>1 were shown in red (heterothermy) and green (homeothermy). The Pika lineage as control was shown in blue. ?: it is likely heterothermic.

To improve our chances of detecting adaptive changes and identifying informative sites within the Chiroptera, we cloned and sequenced exon 3 in an additional 14 bat species. The phylogenetic tree of *Leptin* exon 3 (TrNef+G) was again consistent with multigene phylogenies of mammals and bats [22], [23]. From the exon 3 data, the free-ratio model showed that ω was higher in the ancestral branch to all bats and in the Primate lineage (no synonymous changes) than in other lineages, with ω = 0.581 in the pika lineage. Within the primate lineage, ω ratios of two lineages, the Strepsirrhini (ω = 999.8, N*dN = 3.2, S*dS = 0) and Hominidea (ω = 999.000, N*dN = 6.3, S*dS = 0), are >1. Moreover, the ω ratio of the Yangochiroptera (ω = 974.2, N*dN = 4.6, S*dS = 0) and Rhinolophoidea (ω = 999.0, N*dN = 14.3, S*dS = 0) were greater than those of the Yinpterochiroptea (ω = 0.151) and Pteropodidae (ω = 0.760), respectively (Figure 3). The LRT statistics of the free-ratio model vs. M0 and M3 vs. M0 achieved significance (Table S5). This result is consistent with the analysis based on *Leptin* complete CDS and indicates natural selection drove the evolution of *Leptin* in heterothemic placental mammals, especially in bats (Table S5). Then, the selected pressure on the rhinolophoid lineage was verified by the branch-specific analysis (Table S5). Moreover, data of the free-ratio analysis also indicated the potential of selected pressure (ω>1) on the sub-branchs within pteropodidae, which are homeothermy (Figure 3). Thus, branch-site analysis (Model A) was performed and data showed that the LRT statistic of homeothermic bat lineage (the pteropodid) passed test I (LRT = 10.57, d.f. = 3, P<0.05), but did not pass test II (LRT = 1.40, d.f. = 1, P>0.05), suggesting potential relaxed selection on homeothermic bats (Table S5).

**Figure 4:**
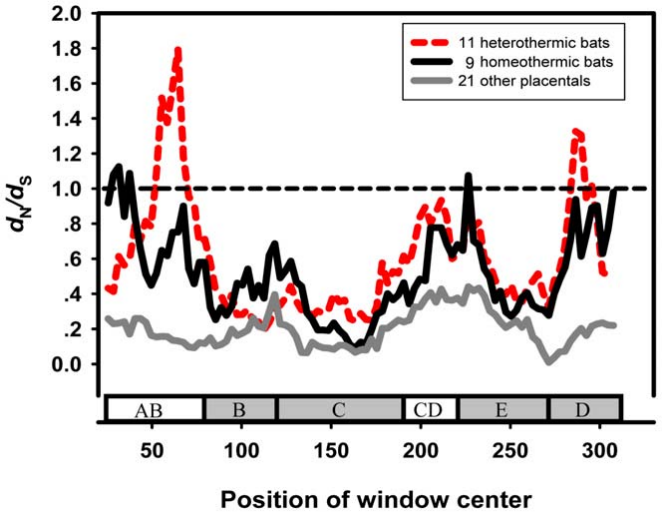
Sliding window analysis of Nei-Gojobori estimates. *d*_N_/*d*_S_ of 21 other placentals, 14 heterothermic bats and 6 hoemothermic bats (Figure 3, excluding *Ursus thibetanus japonicus*, *Microcebus murinus*, *Mus musculus* and the outgroups). In each case is compared to a gene schematic showing the loop (white) and helix (gray) domains, with the window size and step size were fixed at 45 bp and 3 bp. The horizontal broken line identifies ω = 1.

After removing all gaps (12 bp), the alignment of *Leptin* exon 3 consisted of 330 bp encoding 110 amino acids. A sliding window revealed differences in substitution rates among specific groups. The ω values (*d*_N_/*d*_S_) of two groups, heterothermic bats and homeothermic bats, were clearly higher than those of 21 other homeothermic placental mammals (Figure 4). For heterothermic bats, most parts of the *Leptin* exon 3 showed elevated ω ratios, especially in the AB loop and helix D regions, supporting maximum-likelihood based analyses. Moreover, ω values of the 5′ terminal of AB loop and the 5′ terminal helix E in homeothermic bats were higher than in heterothermic bats.

Twenty-nine amino acid variants that were inferred to have large functional effects by multivariate analyses of physicochemical properties, were found in heterothermic bats (mean MAPP score  = 21.9±0.23 (SE); range 7.58–36.58) (Table S6). Of these, seven sites were located in the AB loop, five in the helix B, eight in the helix C, two in the CD loop, one in the helix D, and six in the helix E.

### Lipolysis activity assay of bat Leptin

The substitutions of nucleotides or amino acids may change the spatial structure and affect the function of proteins. Therefore, recombinant GST-Leptin (∼42 kDa) of two bat species, *Miniopterus fuliginosus* (hibernating) and *Rousettus leschenaultii* (homeothermic), was obtained and identified by SDS-PAGE and Western-blot analyses (Figure S3) to compare the functional difference between heterothermic and homeothermic Leptin.

**Figure 5:**
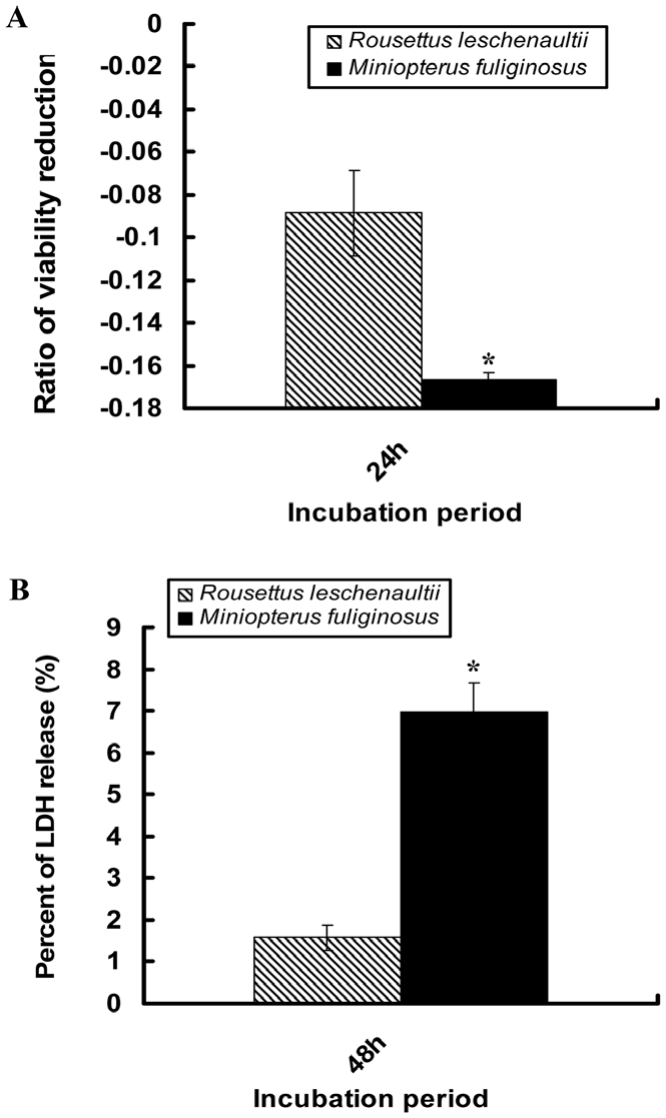
Activity assay of bat Leptin. Relative effect of bat Leptin on viability (MTT) (*A*) and cytotoxicity of 3T3-L1 adipocytes (LDH) (*B*). 3T3-L1 cells were incubated with bat GST-Leptin (10^−6^ M) at 24h or 48h. Cell viability and cytotoxicity were tested by the MTT and LDH assay with four to six replicates for each treatment and experiments were repeated three times. Data are means ± SD. Asterisk indicates significant difference (* P<0.05; *t*-test).

Leptin MTT activity assays, carried out on 3T3-L1 preadipocytes, showed that the number of viable cells treated with heterothermic bat GST-Leptin was less than that of cells incubated with homeothermic bat GST-Leptin (Figure 5A). The proportion of living cells treated with *M. fuliginosus* Leptin decreased by 16.65%, and was significantly lower (P = 0.014) than that for *R. leschenaultii* Leptin (8.85%). An LDH assay (to determine whether the decrease of cell viability was caused by the cytotoxic effect of bat Leptin) showed that after 48 h, 3T3-L1 preadipocytes incubated with 10^−6^ M bat Leptin showed a significantly higher LDH release (P = 0.04; 6.97%) after treatment with *M. fuliginosus* Leptin than those with *R. leschenaultii* Leptin (1.57%; Figure 5B). Consequently, bat Leptin, especially *M. fuliginosus* Leptin, can reduce cell viability and increase LDH release significantly, showing that the Leptin of the heterothemic bat (*M. fuliginosus*) is more active in adipocyte degradation than that of the homeothermic bat (*R. leschenaultii*).

## Discussion

The evolutionary history of mammalian heterothermy remains a controversial subject although it is crucial to the success of a large number of species. In bats, which colonized and are occupying vastly diverse habitats, evolution of heterothermy is especially intriguing given their biogeography and their large range of thermoregulatory abilities. A number of scenarios regarding the evolution of heterothermy in bats have been proposed [1], [14]. If bats evolved in a temperate climate, hibernation is likely to be an ancestral trait that allowed bats to adapt to low environmental temperatures and limited food resources. As these lineages colonized tropical latitudes, the ability of hibernation was lost or was modified to become daily torpor [1]. Alternatively, if bats evolved in the tropics or subtropics and had the ability of daily heterothermy [37], hibernation in temperate species or homeothermy in tropical species are derived states [1]. A third possibility is that ancestral bats were homeothermic, although this seems unlikely given that homeothermy in extant bat taxa is relatively rare. Our data support the view of Lyman [1] that heterothermy in bats is an ancestral trait and was subsequently lost in now homeothermic bats. We provide physiological, molecular and biochemical evidence supporting this interpretation.

Our physiological data show clear differences in the thermoregulatory patterns between tropical/subtropical fruit bats and insectivorous bats when these were subjected to changes in T_a_. Stable T_b_ maintained at low T_a_ in the former demonstrate that these fruit bats are homeothermic, whereas low T_b_ and torpor bouts without arousals for at least 2 days at T_a_ 5°C clearly show that these insectivorous bats have retained the ability to hibernate although they live in tropical/subtropical areas.

In the Chiroptera, most families include heterothermic species, including the New World frugivorous species and some Old World fruit bats [1], [38], [39], [40]. According to the latest molecular phylogenetic data and fossil evidence, the common ancestor of bats originated during the Cenozoic Era around 64 million years ago (Mya) in Laurasia and diverged into two suborders (the Yangochiroptera and Yinpterochiroptera) [22]. Although some extant small species in the Pteropodidae (which originated 24 Mya) still keep the ability of daily torpor [40], most species are considered to be strictly homeothermic consistent with our data (Figure 1), likely because they are restricted to tropical/subtropical climates and have specialized on a diet of fruit [13], [22]. Together with the evolutionary history of the Chiroptera, the chronological distribution of heterothermy in bats, and their current geographic ranges, the most parsimonious assumption is that the ancestors of bats were heterothermic (Figure 2 and Figure S1). Subsequent changes in climate and associated shifts in distribution of species led to the secondary loss of heterothermy in most Old World fruit bats. By comparison, most temperate insectivorous bats and some tropical species have retained their ability to express torpor.

Our molecular phylogenetic analyses with *Leptin* suggest natural selection in heterothermic mammals (Primates and bats). This was achieved by testing the selection pressures upon *Leptin* within placental mammals only, and using the marsupials and monotremes as outgroups because the evolution of heterothermy appears to differ between placental mammals and other mammals [41]. The results of the codon maximum likelihood analysis based on *Leptin* complete CDS and *Leptin* exon 3 were identical and showed that the ω ratios of three lineages, Primates, Hominidea and Strepsirrhini, were >1, consistent with many previous studies [42], [43], [44]. Moreover, our results suggest that natural selection resulted in positive evolution of *Leptin* in the ancestors of bats and also in the heterothermic bats (Figure 3 and Figure S2). In contrast, *Leptin* in the homeothermic bats (pteropodid lineage) seems to be under relaxed selection, according to the LRT statistic of Model A (Table S5). These observations of the evolution of *Leptin* in heterothermic placentals show that this gene can be used as a molecular marker for understanding the evolution of heterothermy in bats.

When the common ancestor of bats originated (around 64 Mya), the worldwide climate was warm; however, long-term cooling occurred in some areas leading to temperate zones when the heterothermic bats (50 Mya-20 Mya) and most homeothermic bats (especially Old World fruit bats, 30 Mya-15 Mya) originated [3], [22]. It is known that the length and depth of heterothermy are affected by body size, and that torpid periods increase with decreasing body size [4], [45]. Most extant hibernating bats are small (5–25 g) and feed on insects [1], [4]. As they require high energy expenditure to maintain a euthermic T_b_, hibernation, as expressed in many temperate bats, is the most efficient way to save energy and survive on limited food [3]. In contrast, large tropical pteropodids (>60 g) appear to be homeothermic, whereas heterothermy seems restricted to a few small New- and Old-World fruit bats [38], [46], [47]. The six species of Pteropodidae (28 Mya–18 Mya) used in our study are homeothermic and tropical/subtropical distribution. Abundant food, a mild climate and a large body size may explain the relaxed selection on *Leptin* of these homeothermic bats. Combining the evolutionary history of homeothermic bats and our molecular data, provides support for our hypothesis that heterothermic thermoregulation was lost in some tropical bats and adapted into hibernation in others. However, to further clearly assess how loss of heterothermy in bats, the heterothermic species of the Pteropodidae should be sampled.

The reconstruction of ancestral states and selection pressure analyses of *Leptin* strongly suggests that the ancestors of bats were heterothermic and that this ability became specialized ultimately resulting in hibernation especially in some temperate bats. Sliding window analysis showed that in heterothermic bats, most bio-functional domains of *Leptin* exon 3 have high ω ratios, especially in the AB loop and helix D (Figure 4). The total 29 amino acid variants specific to heterothermic bats with high MAPP scores were identified in six regions of *Leptin* exon 3 (Table S6). A few studies have analyzed the functional regions and determined the binding sites of Leptin with the receptors, and 10 of 22 binding sites of *Leptin* located in exon 3, including 7 in the functionally significant segment 85–119 [48], [49], [50], [51]. Here, six of 29 sites are in the segment 85–119 and two of them are binding sites indicated that natural selection may affact the intereaction of Leptin with the receptors. In addition, in pika study [21], 17 of 20 sites driven by the cold stress located in five functional regions of exon 3, and four of them were identical with our study (Table S6). More sites and more functional regions located suggessed the selected stress may be stronger in heterothermic bats. These results prove that natural selection may have had a large functional effect on Leptin of heterothermic bats, by stabilizing the conformation and binding with *Leptin* receptors, perhaps for reducing thermal sensitivity [35], [48], [49], [52], [53].

As the sequence is the functional basis of protein, the substitutions of nucleotides or amino acids may lead to the functional change. We were able to validate that amino acid substitutions affect the function of heterothermic bat Leptin, by expressing two Leptin proteins of *Miniopterus fuliginosus* (hibernator) and *Rousettus leschenaultii* (homeothermy) in *E. coli* cells and measuring the lipolytic activity assay *in vitro*. The results of the MTT and LDH assays are consistent and also identical with that of our previous study on Leptin of *Rhinolophus ferrumequinum* and *R. leschenaultii* [54]. Our data show that Leptin in heterothermic bats (*M. fuliginosus and R. ferrumequinum*) is more lipolytic than in homeothermic bats, and there is no significant difference between two heterothermic bats. All these data support the results of physiological and molecular evolutionary analyses. Rewarming from low T_b_ during torpor requires a rapid change in energy supply, and the accurate and efficient mobilization of fat is a key functional component [55]. We showed that Leptin of hibernating bats is more efficient and active than that of homeothermic bats with regard to lipolysis, and this will meet the physiological needs of a rapid rise in energy production. This is critically important because at the low T_b_ during torpor the function of Leptin will be greatly reduced, but a rapid rise in lipolysis for energy production is required during rewarming at the end of a torpor bout. However, the coadaptation between interacting amino acid sites of Leptin and the receptors, and pleiotropy of *Leptin* evolution need to be further explored.

Our physiological study and molecular data, together with the evolutionary history of the Chiroptera, suggest that the ancestors of bats were heterothermic. Following continental drift and the migration of bats, and changes in climate and food habits, bats diverged and became distributed throughout most of the world. Heterothermy became specialized allowing hibernation in predominantly in temperate bats and was lost in some tropical bats, although many bat species still retained the ancestral ability to employ daily torpor. Importantly, the evolution of *Leptin* in bats was driven by natural selection (positive/relaxed selection) and played an important role in the evolution of bat thermoregulation.

## Supporting Information

Text S1 (DOC)

Figure S1 **Ancestral reconstruction of binary data (homeothermy vs. heterothermy.** Branches show homeothermic (white) and heterothermic (black) families. ?: some species of this family are likely heterothermic. For details see Figure 2.
(TIF)

Figure S2 **Gene tree of** ***Leptin*** **.** The phylogenetic gene tree of *Leptin*, based on nucleotide sequences of *Leptin* complete CDS, used for the codon maximum likelihood analysis. The ω ratios and numbers of non-synonymous substitution (N*dN) and synonymous substitution (S*dS) of some specific lineages were shown in bold and in bracket. The scale bar of “0.1” means 0.1 nucleotide substitution per site. [T], torpor; [H], hibernator. Lineages with ω>1 were shown in red and the Pika lineage as control was shown in blue. ?: it is likely heterothermic.
(TIF)

Figure S3 **SDS-PAGE and Western blot analyses of bat GST-Leptins.** GST-Leptin proteins, *R. leschenaultii* (left) and *M.fuliginosus* (right), identified by 10% SDS-PAGE (A)/(B) and Western blot with anti-human-Leptin antibody (C)/(D). GST-Leptin proteins were induced 0 and 4 h by IPTG (0.3 mM) at 30°C.
(TIF)

Table S1 **The geographical distribution and use of torpor in bat species.**
(DOC)

Table S2 **Primer combinations used for amplification of** ***Leptin*** **sequences from 19 bat species.**
(DOC)

Table S3 **Accession number of the** ***Leptin*** **sequences used in this study.**
(DOC)

Table S4 **Likelihood values and parameter estimates for the** ***Leptin*** **complete CDS (32 species, 158 aa).** ω: *d*_N_/*d*_S_ ratio. ℓ: Log-likelihood ratio. Those in parentheses are presented for clarity only but are not free parameters.
(DOC)

Table S5 **Likelihood values and parameter estimates for the** ***Leptin*** **exon 3 (46 species, 110 aa).** ω: *d*_N_/*d*_S_ ratio. ℓ: Log-likelihood ratio. Those in parentheses are presented for clarity only but are not free parameters.
(DOC)

Table S6 **Summary of amino acid variants specifically in heterothermic bats identified by the MAPP analysis.**
(DOC)
