# Correction: Adaptive Evolution of *Leptin* in Heterothermic Bats

**DOI:** 10.1371/annotation/85d2d660-7186-48fa-9e88-5497d837ac7b

**Published:** 2012-01-04

**Authors:** Lihong Yuan, Xudong Zhao, Benfu Lin, Stephen J. Rossiter, Lingjiang He, Xueguo Zuo, Guimei He, Gareth Jones, Fritz Geiser, Shuyi Zhang

There was an error in the funding statement. The correct funding statement is: This work was funded by a grant awarded to SY Zhang under the Key Construction Program of the National "985" Project and "211" Project. LH Yuan is supported by the Guangdong Academy of Sciences Scientific Research Fund (No.qnjj20091) and Guangdong Natural Science Fund (10451026001004389). G Jones, SJ Rossiter and SY Zhang received funding from a Biotechnology and Biological Sciences Research Council (BBSRC) China Partnering Award. The funders had no role in study design, design, data collection and analysis, decision to publish, or preparation of the manuscript. 

