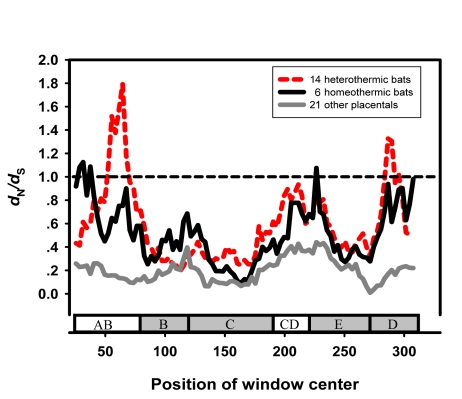# Correction: Adaptive Evolution of *Leptin* in Heterothermic Bats

**DOI:** 10.1371/annotation/374a11a2-e3e8-423d-849f-7dbc1aaaaefb

**Published:** 2012-01-20

**Authors:** Lihong Yuan, Xudong Zhao, Benfu Lin, Stephen J. Rossiter, Lingjiang He, Xueguo Zuo, Guimei He, Gareth Jones, Fritz Geiser, Shuyi Zhang

There is an error in Figure 4. The correct Figure 4 can be viewed here: 

**Figure:**