# Supplementary material for: Adaptive Evolution of Leptin in Heterothermic Bats
Source: PLoS One. 2011 Nov 16;6(11):e27189. doi: 10.1371/journal.pone.0027189 (PMC3217946; doi:10.1371/journal.pone.0027189)
Supplement: Text S1 — (DOC) [file pone.0027189.s001.doc]

**Supporting Information Text**

**Study of thermoregulatory ability**

*Cynopterus sphinx* (n=6), *Eonycteris spelaea* (n=6) and *Scotophilus heathii* (n=6) were collected in Xishuangbanna, Yunnan Province (21°55′N，101°15′E) . *Hipposideros armiger* (n=6) was obtained from Kunming, Yunnan Province (25°04′N，102°42′E). At the time of experiments, *E. spelaea* ranged in body mass from 50.8 to 69.6 g, *C. sphinx* from 44 to 54.3 g, *H. armiger* from 51.9 to 62.2 g and *S. heathii* from 45.9 to 55.8 g. All bats were housed individually in separate cages. *C. sphinx* and *E. spelaea* were supplied with bananas, apples and some honey; *S. heathii* and *H. armiger* were provided with mealworms and water *ad libitum*. The room in which bats were maintained was on a 12:12 light: dark cycle with lights on from 06:00 to 18:00 h. The T_a_ was maintained at 25.5±0.5 ºC. The experiments were conducted in accordance with the guidelines for the National Care and Use of Animals approved by the National Animal Research Authority, in conformance with the National Institutes of Health Guide for Care and Use of Laboratory Animals.

A telemetry T_b_ system (DSI, USA) was used in our study. Bats were surgically implanted with small temperature transmitters (DataSciences ETA10F20; 3.5 g; 2×1×0.7 cm) for recording T_b_. All surgical procedures were conducted under pentobarbital anesthesia (40 mg/kg, i.p.). Transmitters were implanted intraperitoneally through a midline abdominal incision, after which the abdominal incisions in the muscle wall and skin were closed with simple interrupted pattern sutures. When this procedure was completed, the bats remained at T_a_ 25 ºC for a week before experiments began in a climate-controlled cabinet. We defined the onset of torpor as the time when T_b_ was mid-way between the minimum during torpor and the maximum reached in euthermy. The T_b_ data were collected every 30 seconds by Dataquest A.R.T 4.1 (DSI U.S) and daily means ± SE of T_b_ were computed.

During the experiment, the thermoregulatory response of bats to changing T_a_ was quantified. Bats were exposed to T_a_ of 25 ºC, 15 ºC and 5 ºC for 2 days each in sequence; photoperiod was L:D 12:12. These temperatures were selected because they are within the range that is commonly experienced by the species in the wild. At the end of the experiment, the ambient temperature was raised gradually to room temperature.

**Sample collection and template preparation**

We extracted genomic DNA from archived wing membrane biopsies from six species of the family Pteropodidae: *Eidolon helvum*, *Eonycteris spelaea*, *Rousettus leschenaultii*, *Dobsonia viridis*, *Cynopterus sphinx*, *Pteropus giganteus*; and 11 species from other families: *Taphozous melanopogon* (Emballonuridae), *Hipposideros armiger* (Hipposideridae), *Scotophilus heathii*, *Myotis ricketti* (Vespertilionidae), *Chaerephon plicatus*, *Tadarida teniotis* (Molossidae), *Pteronotus parnellii* ([Mormoopidae](http://animaldiversity.ummz.umich.edu/site/accounts/information/Mormoopidae.html)), *Artibeus gnomus*, *Anoura geoffroyi*, *Carollia brevicauda* ([Phyllostomidae](http://animaldiversity.ummz.umich.edu/site/accounts/classification/Phyllostomidae.html)), *Rhinopoma microphyllum* (Rhinopomatidae). In addition, we undertoook RNA extraction from White Adipose tissue (WAT) of *Rhinolophus ferrumequinum* (Rhinolophidae) and *Miniopterus fuliginosus* (Miniopteridae), which were collected during earlier projects on hibernation-related genes. Information on the geographical location of bats analyzed in this study is listed in Table S1.

Genomic DNA was isolated using DNeasy kits (Qiagen) and total RNA by the RNAiso kit (TakaRa). 2 µg of total RNA of each sample was treated with 2 U of RNase-free DNase I (Promega) for 30 min at 37 ºC to avoid genomic DNA contamination, then converted to cDNA by SuperScript III Reverse Transcriptase (Invitrogen) following the manufacturer’s instruction, using 500 ng of random primer, 1 mM dNTP, 2 mM dithiothreitol, 80 U RNase inhibitor (Promega), 1×First-Strand Buffer and 400 U SuperScrip III Reverse Transcriptase in a 50 µl reaction mixture. The cDNA was stored at -20 ºC until used for PCR amplification.

**Amplification and sequencing of *Leptin***

The *Leptin* coding-domain sequence (CDS) comprises two exons: exon 2 (144 bp) and exon 3 (357 bp) separated by intron 2. Exon 2 codes for 48 amino acids with a signal sequence of 21 residues, while exon 3 codes for 119 amino acids and accounts for more than 80% (119/146 amino acids) of the mature protein. To isolate the complete *Leptin* CDS of *R. ferrumequinum* and *M. fuliginosus*, the first-strand cDNA was used as a template. For PCR, 30 cycles (94 °C for 2 min, 54 °C for 30 s, and 72 °C for 2 min) were performed with the degenerated primers (PF1: 5’ AAG AAG MMS ATC CYR GGR AGG AAA ATG 3’ and PR1: 5’ WGR CCT TYR ARR CYT CA GCA YYC AG 3’) and Taq Polymerase (TaKaRa). In addition, two primer pairs were used to amplify two overlapping regions with gDNA as the template, PF1/PR2 (PR2: 5’ CTG AGC YRT CYY TGC TTC TGA CCA CCT A 3’) to obtain exon 2 to intron 2, PF2 (PF2: 5’ RTC TCC TGR YCA YTG TGG TRG TCT SAC AGT 3’), PR1 to obtain intron 2 to exon 3, and to obtain the whole *Leptin* CDS of three bat species (*M. ricketti*, *E. spelaea* and *R. leschenaultii*).

As exon 3 accounts for over two thirds of the CDS, we surveyed this exon in an additional 14 bat species, using gDNA as the template with the following primer pairs: PF2 and PR1 for *D. viridis*, *C. sphinx*, *T. melanopogon* and *S. heathii*, PF3 (5’ RTC TCC TRA YCA YTG TGG RTG TCT G 3’) and PR3 (5’ TGG CCT TTA ARG CTT CAG CAC CCA G 3’) for *E. helvum* and *T. teniotis*, and PF4 (5’ GGC AGC YAM CTR GGY RCA ASA AAT AA 3’) and PR3 for *P. giganteus*, *H. armiger*, *C. plicatus*, *P. parnellii*, *A. gnomus*, *A. geoffroyi*, *C. brevicauda* and *R. microphyllum*.

The PCR products from cDNA and gDNA were ligated into pMD-19T vector (TaKaRa) by T4 DNA ligase (TaKaRa) in 16 °C for four hours, and clones were sequenced using a Big Dye Terminator on an ABI 3730 DNA sequencer. Detailed information on primer combinations and annealing temperatures are shown in Table S2.

**Database searches**

In addition to sequencing the *Leptin* complete CDS in five bat species and the exon 3 of *Leptin* in another 14 bat species, we also obtained the complete *Leptin* sequences of a further 27 mammal species from the NCBI Nucleotide collection (nr) and Whole-genome shotgun (wgs) databases (Pruitt, Tatusova, and Maglott 2005). These comprised six primates: *Homo sapiens* (BC060830), *Pan troglodytes* (XM_519353), *Pongo abelii* (ABGA01381057), *Macaca mulatta* (U58492), *Otolemur garnettii* (AAQR01061931) and *Microcebus murinus* (ABDC01008790); two rodents: *Rattus norvegicus* (NM_013076) and *Mus musculus* (BC125245); eight lagomorphs: *Lepus oiostolus* (DQ983190), *Oryctolagus cuniculus* (DQ983191), *Ochotona dauurica bedfordi* (EF091860), *Ochotona annectens* (EF091862), *Ochtona nubrica* (EF091861), *Ochotona curzoniae* (DQ268537), *Ochotona cansus cansus* (EF091863) and *Ochotona princeps* (AAYZ01273892); three artiodactyls: *Sus scrofa* (NM_213840), *Bos taurus* (BT020625) and *Capra hircus* (EF583947); one perissodactyl: *Equus caballus* (XM_001502622); three carnivores: *Felis catus* (NM_001009850), *Ursus thibetanus japonicus* (AB255164) and *Canis lupus familiaris* (AB020986); one bat: *Myotis lucifugus* (AAPE01536993); one proboscidean: *Loxodonta africana* (AAGU01099489/AAGU01099488); one marsupial: *Monodelphis domestica* (AAFR03031913), and one monotreme: *Ornithorhynchus anatinus* (AAPN01131030). Details are provided in Table S3.

**Evolutionary analyses**

To identify the variable selective pressures in the *Leptin*, both across the tree and along specific lineages, we used the CODEML program in the PAML 3.15 package. Four codon substitution models were performed: a free-ratio model to evaluate the potential for natural selection in any one branch of the tree, site-specific models to test the selective pressure among sites, a branch-specific model (two-ratio) to test for changes of selective pressure in specific lineages, and a branch-site model (Model A) to distinguish positive or relaxed selection on specific lineages (Yang 2007). (Yang 2007). Likelihood ratio test (LRT) statistics were calculated as twice the difference in maximum likelihood values (2*Δℓ*) between nested models, and significance determined using the *X^2^* distribution. We also included published sequence data from the lagomorph group of pikas, in which molecular evolution of *Leptin* has previously been shown to be linked to cold environmental stresses (Yang et al. 2008).

The free-ratio model assumes an independent ω ratio for each branch of a topology and the LRT statistic was calculated by free-ratio model vs. M0 (one-ratio model), which assumes the same ω ratio for all lineages in the gene (and all branches in the phylogeny). The site-specific models allow the ω ratio to vary among sites but fix one ω ratio in all lineages. The Nearly Neutral model (M1a) estimates two ω values (0<ω_0_<1, ω_1_=1); the positive selection model (M2a) adds an additional ω value to M1a, which allows ω_2_ greater than one, if present; the discrete model (M3) extends from M0 and has two unconstrained ω values (ω_1_ and ω_2_); M8 (beta & ω model) and M8a (fix omega=1) are extended from M7, which constrains ω ratio smaller than one following the β distribution, and takes into account possible positively selected (PS) sites (Yang 1997). Three pairs of models were compared (M2a versus M1a, M3 versus M0, and M8 versus M8a) and significance assessed based on LRTs. If the ω ratio is variable among the specific lineages, the branch-specific model (two-ratio) was implemented to test for changes in selective pressure in specific lineages. This approach assigned two ω ratios for the foreground (ω_1_) and background (ω_0_), and allows for variable ω ratios among branches but invariable ω ratios in sites in the tree (Yang and Nielsen 2002). Then, the branch-specific model was compared with M0 to perform a LRT test. To assess whether the inferred gain or loss of heterothermy in bats was associated with changes in selection pressure (positive or relaxed selection), we used the branch-site model (Model A). This model assigns three ω ratios (0<ω_0_<1, ω_1_=1, and ω_2_ is variable among sites), and two LRT statistics were obtained (test I, Model A vs. M1a; test II, Model A vs. Model A’, fix_omega=1). If the LRT statistic of test I reached significance, whereas that of test II did not, then relaxed selection was inferred (Yang 1997).

**GST-*Leptin* expression and Western-blot assay**

*Leptin* coding sequences of the two bats, excluding the first 21 amino acids signature domain were amplified by PCR, and two pairs of primers with BamH I/Sal I restriction sites were used and underlined in primer pairs (5’CC GGA TCC GCG CCC ATC CAA AAA GTC 3’/ 5’ CC GTC GAC TCA GCA TCC AGG GCT G 3’for *M. fuliginosus*; 5’CC GGA TCC GTG CCC ATC CAA AAA GTC CAG GAT GAC 3’/ 5’ CC GTC GAC TCA GCA TCC AGG GCT GA 3’for *R. leschenaultii*). PCR products were cleaned, and digested with BamH I and Sal I restriction enzymes (TakaRa) prior to ligation to PGEX-4T-2 vector (Invitrogen) by T4 DNA ligase (TakaRa). The ligation products were transferred to DH5α *E. coli* cells (TakaRa) and positive clones were sequenced to confirm the target sequence. GST-*Leptin* recombinant plasmid was purified, transformed into BL21 *E. coli* cells (TakaRa) and induced by 5-bromo-4-chloro-3-indolyl-phosphate (IPTG) (TakaRa) to obtain the recombinant Leptin proteins. SDS-PAGE and Western blots were carried out with Coomassie Brilliant Blue R-250 staining and anti-human-Leptin antibody (rabbit polyclonal antibody, Santa Cruz Biotechnology), respectively, to determine the purity and monomer content of proteins. Finally, the recombinant proteins were purified by GST Fusion Protein Purification Kit (GenScript) and concentrated with Amicon Ultra-4 Centrifugal Filter Devices (MILIPORE).

**References:**

Grasso, P., M. C. Leinung, S. P. Ingher, and D. W. Lee. 1997. In vivo effects of leptin-related synthetic peptides on body weight and food intake in female ob/ob mice: localization of leptin activity to domains between amino acid residues 106-140. Endocrinology **138**:1413-1418.

Grasso, P., D. W. White, L. A. Tartaglia, M. C. Leinung, and D. W. Lee. 1999. Inhibitory effects of leptin-related synthetic peptide 116-130 on food intake and body weight gain in female C57BL/6J ob/ob mice may not be mediated by peptide activation of the long isoform of the leptin receptor. Diabetes **48**:2204-2209.

Hiroike, T., J. Higo, H. Jingami, and H. Toh. 2000. Homology modeling of human leptin/leptin receptor complex. Biochem Biophys Res Commun **275**:154-158.

Lee, G. H., R. Proenca, J. M. Montez, K. M. Carroll, J. G. Darvishzadeh, J. I. Lee, and J. M. Friedman. 1996. Abnormal splicing of the leptin receptor in diabetic mice. Nature **379**:632-635.

Pruitt, K. D., T. Tatusova, and D. R. Maglott. 2005. NCBI Reference Sequence (RefSeq): a curated non-redundant sequence database of genomes, transcripts and proteins. Nucleic Acids Res **33**:D501-504.

Yang, J., Z. L. Wang, X. Q. Zhao, P. Wang de, L. Qi de, B. H. Xu, Y. H. Ren, and H. F. Tian. 2008. Natural selection and adaptive evolution of leptin in the ochotona family driven by the cold environmental stress. PLoS ONE **3**:e1472.

Yang, Z. 2007. PAML 4: phylogenetic analysis by maximum likelihood. Mol Biol Evol **24**:1586-1591.

Yang, Z. 1997. PAML: a program package for phylogenetic analysis by maximum likelihood. Comput Appl Biosci **13**:555-556.

Zhang, F., M. B. Basinski, J. M. Beals, S. L. Briggs, L. M. Churgay, D. K. Clawson, R. D. DiMarchi, T. C. Furman, J. E. Hale, H. M. Hsiung, B. E. Schoner, D. P. Smith, X. Y. Zhang, J. P. Wery, and R. W. Schevitz. 1997. Crystal structure of the obese protein leptin-E100. Nature **387**:206-209.
