# Supplementary figures and images for: Adaptive Evolution of Leptin in Heterothermic Bats
Source: PLoS One. 2011 Nov 16;6(11):e27189. doi: 10.1371/journal.pone.0027189 (PMC3217946; doi:10.1371/journal.pone.0027189)

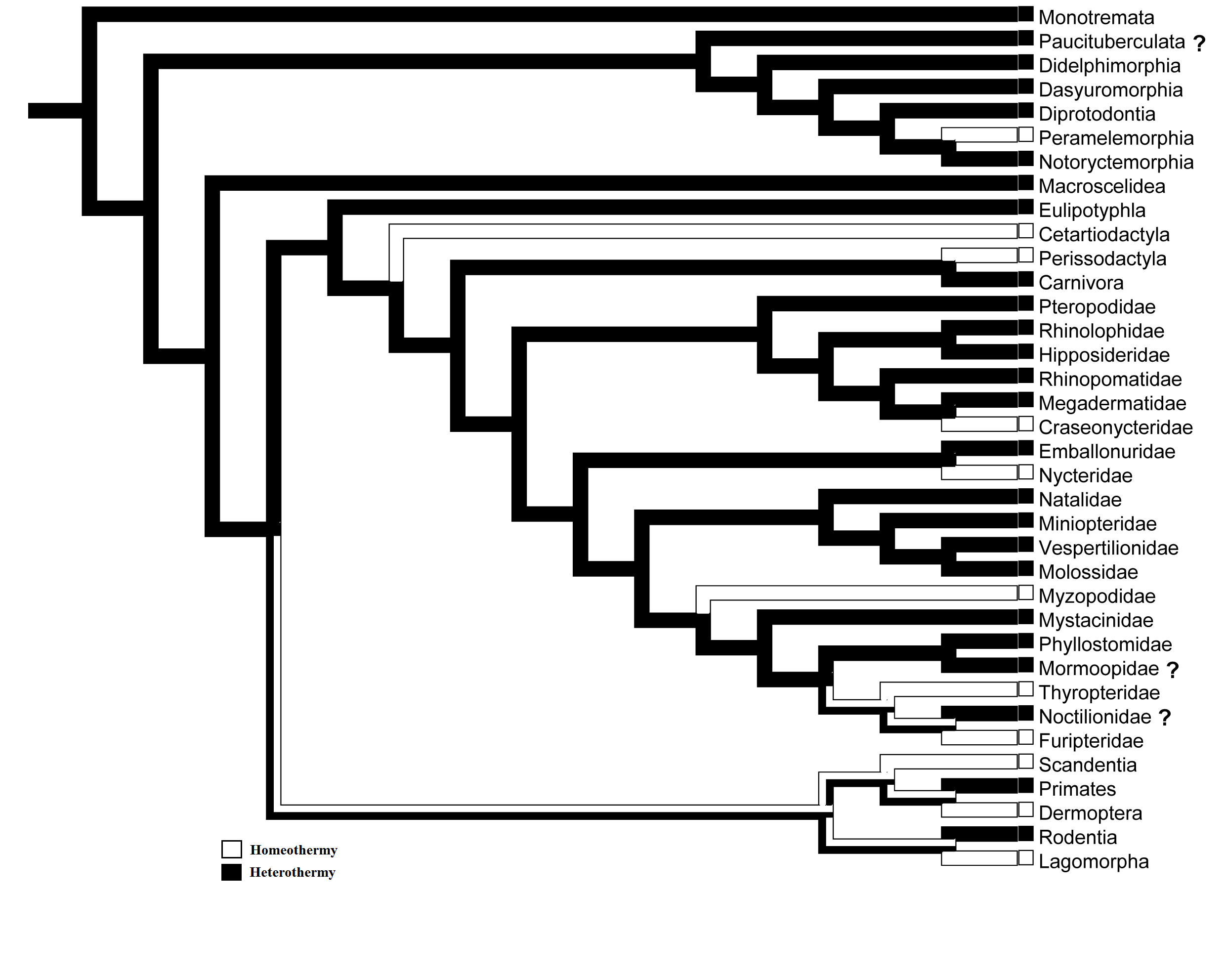

Supplement: Figure S1 — Ancestral reconstruction of binary data (homeothermy vs. heterothermy. Branches show homeothermic (white) and heterothermic (black) families. ?: some species of this family are likely heterothermic. For details see Figure 2. (TIF) [file pone.0027189.s002.tif]

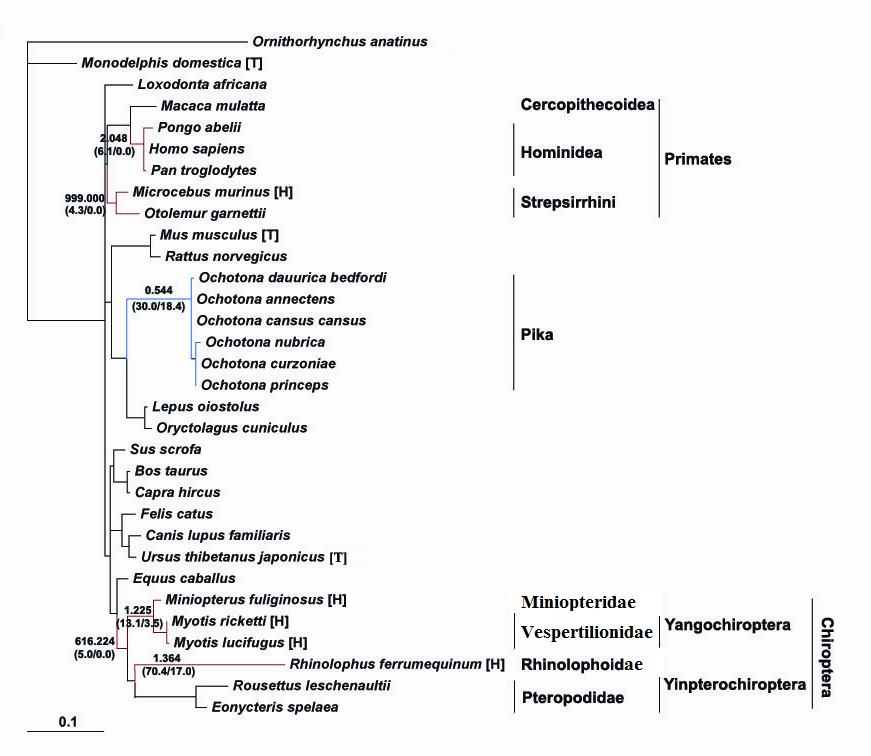

Supplement: Figure S2 — Gene tree of Leptin . The phylogenetic gene tree of Leptin, based on nucleotide sequences of Leptin complete CDS, used for the codon maximum likelihood analysis. The ω ratios and numbers of non-synonymous substitution (N*dN) and synonymous substitution (S*dS) of some specific lineages were shown in bold and in bracket. The scale bar of “0.1” means 0.1 nucleotide substitution per site. [T], torpor; [H], hibernator. Lineages with ω>1 were shown in red and the Pika lineage as control was shown in blue. ?: it is likely heterothermic. (TIF) [file pone.0027189.s003.tif]

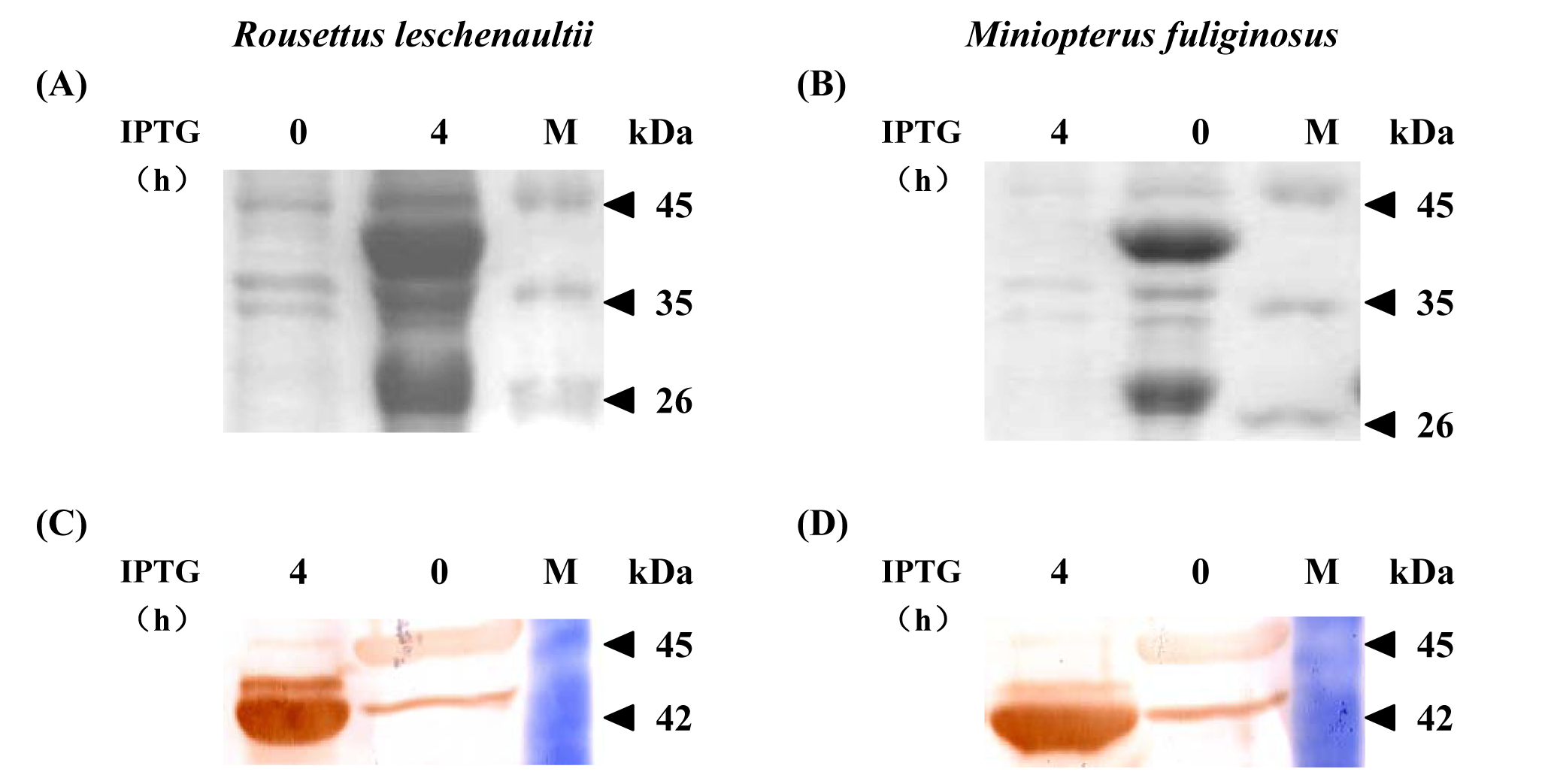

Supplement: Figure S3 — SDS-PAGE and Western blot analyses of bat GST-Leptins. GST-Leptin proteins, R. leschenaultii (left) and M.fuliginosus (right), identified by 10% SDS-PAGE (A)/(B) and Western blot with anti-human-Leptin antibody (C)/(D). GST-Leptin proteins were induced 0 and 4 h by IPTG (0.3 mM) at 30°C. (TIF) [file pone.0027189.s004.tif]
