# Supplementary material for: Adaptive Evolution of Leptin in Heterothermic Bats
Source: PLoS One. 2011 Nov 16;6(11):e27189. doi: 10.1371/journal.pone.0027189 (PMC3217946; doi:10.1371/journal.pone.0027189)
Supplement: Table S1 — The geographical distribution and use of torpor in bat species. (DOC) [file pone.0027189.s005.doc]

**Table S1. The geographical distribution and use of heterothermy in bat species.**

| Species | Geographical location | **Thermal Physiology** |
| --- | --- | --- |
| **Family** [**Molossidae**](http://animaldiversity.ummz.umich.edu/site/accounts/classification/Molossidae.html) |  |  |
| *Chaerephon plicatus* (Wrinkle-lipped free-tailed bat) | Guiping, Guangxi, China (23°11.7′N, 110°12.5′E) | Hibernation |
| *Tadarida teniotis* (European free-tailed bat) | Yiliang, Yunnan, China (25°4′N, 103°22.8′E) | Hibernation |
| **Family Vespertilionidae** |  |  |
| *Myotis ricketti* (Rickett's big-footed bat) | Beijing, China (39°48′N, 115°42′E) | Hibernation |
| *Scotophilus heathii* (Greater Asiatic yellow house bat) | Ningming, Guangxi, China (22°07′N, 107°7′E) | Hibernation |
| **Family Miniopteridae** |  |  |
| *Miniopterus fuliginosus* | Liuan, Anhui, China (31°32′N, 116°08′E) | Hibernation |
| **Family** [**Mormoopidae**](http://animaldiversity.ummz.umich.edu/site/accounts/information/Mormoopidae.html) |  |  |
| *Pteronotus parnellii* (Parnell's Mustached bat) | French Guiana  ( 4°072′N, 52°732′W) | Torpor ? |
| **Family** [**Phyllostomidae**](http://animaldiversity.ummz.umich.edu/site/accounts/classification/Phyllostomidae.html) |  |  |
| *Artibeus gnomus* (Dwarf fruit-eating bat) | French Guiana  ( 4°072′N, 52°732′W) | Torpor ? |
| *Anoura geoffroyi* (Geoffroy's tailless bat) | French Guiana  ( 4°072′N, 52°732′W) | Torpor ? |
| *Carollia brevicauda*  (silky short-tailed bat) | French Guiana  ( 4°072′N, 52°732′W) | Torpor ? |
| **Family Emballonuridae** |  |  |
| *Taphozous melanopogon* (black-bearded tomb bat) | Guilin, Guangxi, China (25°16.2′N, 111°21.1′E) | Hibernation |
| **Family Hipposideridae** |  |  |
| *Hipposideros armiger* (Great roundleaf bat) | Chizhou, Anhui, China (30°20′N, 117°50′E) | Hibernation |
| **Family Rhinolophidae** |  |  |
| *Rhinolophus ferrumequinum* (Greater horseshoe bat) | Beijing, China (39°48′N, 115°42′E) | Hibernation |
| **Family Rhinopomatidae** |  |  |
| *Rhinopoma microphyllum* (Greater mouse-tailed bat) | Nabatieh, Lebanon ( 33°10′N, 35°39′E) | Torpor/Hibernation |
| **Family Pteropodidae** |  |  |
| *Rousettus leschenaultii* (Leschenault's rousette) | Menglun, Yunnan, China (21°59.2′N, 101°21.4′E) | Homeothermy |
| *Eonycteris spelaea* (Lesser dawn bat/Long-tongued dawn fruit bats) | Xishuangbanna, Yunnan, China (21°55.3′N, 101°15′E) | Homeothermy |
| *Dobsonia viridis* (Greenish naked-backed fruit bat) | Buton | Homeothermy |
| *Eidolon helvum* (Straw-colored fruit bat) | Lubee Bat Conservancy | Homeothermy |
| *Pteropus giganteus* (Indian flying fox) | Lubee Bat Conservancy | Homeothermy |
| *Cynopterus sphinx* (Greater short-nosed fruit bat) | Guangzhou, Guangdong, China (23°08′N, 115°15′E) | Homeothermy |

?: it is likely heterothermic, but no references.
