# Supplementary material for: Adaptive Evolution of Leptin in Heterothermic Bats
Source: PLoS One. 2011 Nov 16;6(11):e27189. doi: 10.1371/journal.pone.0027189 (PMC3217946; doi:10.1371/journal.pone.0027189)
Supplement: Table S2 — Primer combinations used for amplification of Leptin sequences from 19 bat species. (DOC) [file pone.0027189.s006.doc]

**Table S2. Primer combinations used for amplification of *Leptin* sequences from 19 bat species.**

| Primer combinations | Primer sequence | Annealing temperature | Template | Species | Products |
| --- | --- | --- | --- | --- | --- |
| PF1/PR1 | 5’ AAG AAG MMS ATC CYR GGR AGG AAA ATG 3’  5’ WGR CCT TYR ARR CYT CA GCA YYC AG 3’ | 54°C | cDNA | *Rhinolophus ferrumequinum*  *Miniopterus fuliginosus* | Full CDS |
| PF1/PR2  PF2/PR1 | 5’ AAG AAG MMS ATC CYR GGR AGG AAA ATG 3’  5’ CTG AGC YRT CYY TGC TTC TGA CCA CCT A 3’  5’ RTC TCC TGR YCA YTG TGG TRG TCT SAC AGT 3’  5’ WGR CCT TYR ARR CYT CA GCA YYC AG 3’ | 54°C  54°C | gDNA | *Myotis ricketti*  *Eonycteris spelaea*  *Rousettus leschenaultii* | Full CDS |
| PF2/PR1 | 5’ RTC TCC TGR YCA YTG TGG TRG TCT SAC AGT 3’  5’ WGR CCT TYR ARR CYT CA GCA YYC AG 3’ | 54°C | gDNA | *Dobsonia viridis*  *Cynopterus sphinx*  *Taphozous melanopogon*  *Scotophilus heathii* | Exon 3 |
| PF3/PR3 | 5’ RTC TCC TRA YCA YTG TGG RTG TCT G 3’  5’ TGG CCT TTA ARG CTT CAG CAC CCA G 3’ | 53.6°C | gDNA | *Eidolon helvum*  *Tadarida teniotis* | Exon 3 |
| PF4/PR3 | 5’ GGC AGC YAM CTR GGY RCA ASA AAT AA 3’  5’ TGG CCT TTA ARG CTT CAG CAC CCA G 3’ | 53.6°C | gDNA | *Pteropus giganteus*  *Hipposideros armiger*  *Chaerephon plicatus*  *Pteronotus parnellii*  *Artibeus gnomus*  *Anoura geoffroyi*  *Carollia brevicauda*  *Rhinopoma microphyllum* | Exon 3 |

NOTE—The start codon and stop codon are underlined.
