# Supplementary material for: Adaptive Evolution of Leptin in Heterothermic Bats
Source: PLoS One. 2011 Nov 16;6(11):e27189. doi: 10.1371/journal.pone.0027189 (PMC3217946; doi:10.1371/journal.pone.0027189)
Supplement: Table S3 — Accession number of the Leptin sequences used in this study. (DOC) [file pone.0027189.s007.doc]

**Table S3. Accession number of the *Leptin* sequences used in this study.**

| **Species** | **GenBank Accession Numbers** | **Thermal Physiology** |
| --- | --- | --- |
| *Homo sapiens* | BC060830 | Homeothermy |
| *Pan troglodytes* | XM_519353 | Homeothermy |
| *Pongo abelii* | ABGA01381057 | Homeothermy |
| *Macaca_mulatta* | U58492 | Homeothermy |
| *Microcebus murinus* | ABDC01008790 | Hibernation |
| *Otolemur garnettii* | AAQR01061931 | Homeothermy |
| *Mus musculus* | BC125245 | Torpor |
| *Rattus norvegicus* | NM_013076 | Homeothermy |
| *Lepus oiostolus* | DQ983190 | Homeothermy |
| *Oryctolagus cuniculus* | DQ983191 | Homeothermy |
| *Ochotona dauurica bedfordi* | EF091860 | Homeothermy |
| *Ochotona annectens* | EF091862 | Homeothermy |
| *Ochtona nubrica* | EF091861 | Homeothermy |
| *Ochotona curzoniae* | DQ268537 | Homeothermy |
| *Ochotona cansus cansus* | EF091863 | Homeothermy |
| *Ochotona princeps* | AAYZ01273892 | Homeothermy |
| *Sus scrofa* | NM_213840 | Homeothermy |
| *Bos Taurus* | BT020625 | Homeothermy |
| *Capra hircus* | EF583947 | Homeothermy |
| *Equus caballus* | XM_001502622 | Homeothermy |
| *Felis catus* | NM_001009850 | Homeothermy |
| *Canis lupus familiaris* | AB020986 | Homeothermy |
| *Ursus thibetanus japonicus* | AB255164 | Torpor |
| *Chaerephon plicatus* | GU230836 | Hibernation |
| *Tadarida teniotis* | GU230839 | Hibernation |
| *Myotis ricketti* | GU230846 | Hibernation |
| *Myotis lucifugus* | AAPE01536993 | Hibernation |
| *Miniopterus fuliginosus* | GU230844 | Hibernation |
| *Scotophilus heathii* | GU230843 | Hibernation |
| *Artibeus gnomus* | GU230832 | Torpor ? |
| *Anoura geoffroyi* | GU230833 | Torpor ? |
| *Carollia brevicauda* | GU230829 | Torpor ? |
| *Pteronotus parnellii* | GU230831 | Torpor ? |
| *Taphozous melanopogon* | GU230842 | Hibernation |
| *Hipposideros armiger* | GU230835 | Hibernation |
| *Rhinolophus ferrumequinum* | GU230845 | Hibernation |
| *Rhinopoma microphyllum* | GU230830 | Hibernation/Torpor |
| *Rousettus leschenaulti* | GU230847 | Homeothermy |
| *Eonycteris spelaea* | GU230848 | Homeothermy |
| *Dobsonia viridis* | GU230840 | Homeothermy |
| *Eidolon helvum* | GU230838 | Homeothermy |
| *Pteropus giganteus* | GU230837 | Homeothermy |
| *Cynopterus sphinx* | GU230841 | Homeothermy |
| *Loxodonta africana* | AAGU01099489/AAGU01099488 | Homeothermy |
| *Monodelphis domestica* | AAFR03031913 | Hibernation/Torpor |
| *Ornithorhynchus anatinus* | AAPN01131030 | Homeothermy |

NOTE—The Leptin coding domain sequences of 27 species were downloaded from NCBI Nucleotide collection (nr) and Whole-genome shotgun (wgs) databases; other Leptin coding domain sequences of 19 bat species were cloned in this study. ?: it is likely heterothermic.
