# Supplementary material for: Adaptive Evolution of Leptin in Heterothermic Bats
Source: PLoS One. 2011 Nov 16;6(11):e27189. doi: 10.1371/journal.pone.0027189 (PMC3217946; doi:10.1371/journal.pone.0027189)
Supplement: Table S4 — Likelihood values and parameter estimates for the Leptin complete CDS (32 species, 158 aa). ω: d N/d S ratio. ℓ: Log-likelihood ratio. Those in parentheses are presented for clarity only but are not free parameters. (DOC) [file pone.0027189.s008.doc]

**Table S4. Likelihood values and parameter estimates for the *Leptin* complete CDS (32 species, 158 aa).**

| Model/Likelihood ratio test (LRT) | Estimates of Parameters | ℓ | 2*Δℓ* | df | *P*-value | Positively selected sites |
| --- | --- | --- | --- | --- | --- | --- |
| M0: one-ratio | ω=0.296 | -4568.20 | - | - | - | None |
| Free-ratio | variable ω by branch | -4503.27 | - | - | - | Not allowed |
| Site-specific models |  |  |  |  |  |  |
| M1a: NearlyNeutral (K=2) | *p*_0_=0.688, (*p*_1_=0.312) | -4490.58 | - | - | - | Not allowed |
| M2a: Positive selection (K=3) | *p*_0_=0.684, *p*_1_=0.308, (***p*_2_=0.008**) (ω_0_=0.139, ω_1_=1.000), **ω_2_=4.272** | -4488.99 | - | - | - | 8G, 25Q |
| M3: discrete (K=3) | *p*_0_=0.508, *p*_1_=0.386, (***p*_2_=0.106**) ω_0_=0.073, ω_1_=0.454, **ω_2_=1.141** | -4478.06 | - | - | - | 2H, 4G, 5T, 8G (P<0.01), 15Y, 19V (P<0.05), 25Q (P<0.05), 50S, 87S, 94I, 112F, 119W, 121S, 124E, 153Q |
| M8a: fix omega=1 | *P*=0.862, *q*=2.868 | -4478.99 | - | - | - | None |
| M8: beta&ω>1 | *p_0_*=0.9872, *p*=0.6320, *q*=1.410, (***p*_1_=0.013**), **ω=2.913** | -4478.18 | - | - | - | 8G, 19V, 25Q |
| Branch-specific model (Two-ratio) |  |  |  |  |  |  |
| Chiroptera lineage | ω_0_= 0.2813, **ω_1_= 999.0000** | -4560.80 | - | - | - | Not allowed |
| Yangochiropteran lineage | ω_0_= 0.2787, **ω_1_= 1.2506** | -4560.73 | - | - | - | Not allowed |
| Rhinolophoid lineage | ω_0_= 0.2558, **ω_1_= 1.3438** | -4549.78 | - | - | - | Not allowed |
| Strepsirrhini lineage | ω_0_= 0.2827, **ω_1_= 2.9003** | -4562.42 | - | - | - | Not allowed |
| Hominid lineage | ω_0_= 0.2817, **ω_1_= 2.4390** | -4561.41 | - | - | - | Not allowed |
| LRT of variable ω values among sites |  |  |  |  |  |  |
| M0 vs. M3 |  | - | 180.28 | 4 | <0.001 | - |
| M1a vs. M2a |  | - | 3.19 | 2 | >0.05 | - |
| M8a vs. M8 |  | - | 1.62 | 1 | >0.05 | - |
| LRT of variable ω values among branches |  |  |  |  |  | - |
| Free-ratio vs. M0 |  | - | 129.86 | 60 | <0.001 | - |
| LRT of ω at specific lineages (one ratio vs. two ratio) |  |  |  |  |  | - |
| Chiroptera lineage |  | - | 14.80 | 1 | <0.001 | - |
| Yangochiropteran lineage |  | - | 14.94 | 1 | <0.001 | - |
| Rhinolophoid lineage |  | - | 36.84 | 1 | <0.001 | - |
| Strepsirrhini lineage |  | - | 11.56 | 1 | <0.001 | - |
| Hominid lineage |  | - | 13.58 | 1 | <0.001 | - |

ω: *d*_N_/*d*_S_ ratio. ℓ: Log-likelihood ratio. Those in parentheses are presented for clarity only but are not free parameters. Positively selected sites are located with Human *Leptin* complete CDS as reference and gaps are removed.
