# Supplementary material for: Adaptive Evolution of Leptin in Heterothermic Bats
Source: PLoS One. 2011 Nov 16;6(11):e27189. doi: 10.1371/journal.pone.0027189 (PMC3217946; doi:10.1371/journal.pone.0027189)
Supplement: Table S5 — Likelihood values and parameter estimates for the Leptin exon 3 (46 species, 110 aa). ω: d N/d S ratio. ℓ: Log-likelihood ratio. Those in parentheses are presented for clarity only but are not free parameters. (DOC) [file pone.0027189.s009.doc]

**Table S5. Likelihood values and parameter estimates for the *Leptin* exon 3 (46 species, 110 aa).**

| Model/Likelihood ratio test (LRT) | Estimates of Parameters | ℓ | 2*Δℓ* | df | P-value | Positively selected sites |
| --- | --- | --- | --- | --- | --- | --- |
| M0: one-ratio | ω=0.294 | -4313.38 | - | - | - | None |
| Free-ratio | variable ω by branch | -4223.34 | - | - | - | Not allowed |
| Site-specific models |  |  |  |  |  |  |
| M1a: NearlyNeutral (K=2) | *p*_0_=0.684, (*p*_1_=0.316) | -4269.73 | - | - | - | Not allowed |
| M2a: Positive selection (K=3) | *p*_0_=0.684, *p*_1_=0.255, (*p*_2_=0.061) (ω_0_=0.175, ω_1_=1.000), ω_2_=0.999 | -4269.73 | - | - | - | None |
| M3: discrete (K=3) | *p*_0_=0.284, *p*_1_=0.399, (*p*_2_=0.317) ω_0_=0.038, ω_1_=0.250, ω_2_=0.643 | -4247.48 | - | - | - | None |
| M8a: fix omega=1 | *P*=0.767, *q*=1.640 | -4247.68 | - | - | - | None |
| M8: beta&ω>1 | *p_0_*=1.000, *p*=0.772, *q*=1.655, (*p*_1_=0.000), ω=1.000 | -4247.70 | - | - | - | None |
| Branch-specific model (Two-ratio) |  |  |  |  |  |  |
| Chiroptera lineage | ω_0_= 0.2912, **ω_1_= 1.8494** | -4312.54 | - | - | - | Not allowed |
| Yangochiroptera lineage | ω_0_= 0.2914, **ω_1_= 1.3581** | -4312.43 | - | - | - | Not allowed |
| Rhinolophoidea lineage | ω_0_= 0.2830, **ω_1_= 999.0000** | -4305.08 | - | - | - | Not allowed |
| Primate lineage | ω_0_= 0.2942, ω_1_= 0.2950 | -4313.38 | - | - | - | Not allowed |
| Strepsirrhini lineage | ω_0_= 0.2942, ω_1_= 0.2950 | -4313.11 | - | - | - | Not allowed |
| Hominid lineage | ω_0_= 0.2925, ω_1_= 0.7199 | -4309.52 | - | - | - | Not allowed |
| Branch-site models |  |  |  |  |  |  |
| Model A | *p*_0_= 0.3013, *p*_1_= 0.1339, ( *p*_2_+*p*_3_= 0.5648) (ω_0_= 0.1679, ω_1_= 1.0000), **ω_2_=1.3552** | -4266.66 | - | - | - | 3V, 7K, 16P, 19P, 41P, 44N, 61V, 65K, 75L, 85A |
| Model A’ | *p*_0_= 0.2376, *p*_1_= 0.1059, ( *p*_2_+*p*_3_= 0.6565) (ω_0_= 0.1679, ω_1_= 1.0000), ω_2_=1.0000 | -4266.72 | - | - | - | None |
| LRT of variable ω values among sites |  |  |  |  |  |  |
| M0 vs. M3 | - | - | 131.8 | 4 | <0.001 | - |
| M1a vs. M2a | - | - | 0 | 2 | >0.05 | - |
| M8a vs. M8 | - | - | 0.36 | 1 | >0.05 | - |
| LRT of variable ω values among branches |  |  |  |  |  |  |
| Free-ratio vs. M0 | - | - | 129.86 | 60 | <0.001 | - |
| LRT of ω at specific lineages (one ratio vs. two ratio) |  |  |  |  |  |  |
| Chiroptera lineage | - | - | 1.68 | 1 | >0.05 | - |
| Yangochiroptera lineage | - | - | 1.9 | 1 | >0.05 | - |
| Rhinolophoidea lineage | - | - | 16.6 | 1 | <0.001 | - |
| Primate lineage | - | - | 0 | 1 | >0.05 | - |
| Strepsirrhini lineage | - | - | 0.54 | 1 | >0.05 | - |
| Hominid lineage | - | - | 7.72 | 1 | <0.01 | - |
| LRT of variable ω along homeothermic bat lineages |  |  |  |  |  |  |
| Model 1a vs. Model A (test I) |  |  | 5.8 | 1 | <0.05 | - |
| Model A’ vs. Model A (test II) |  |  | 0.14 | 1 | >0.05 | - |

ω: *d*_N_/*d*_S_ ratio. ℓ: Log-likelihood ratio. Those in parentheses are presented for clarity only but are not free parameters. Positively selected sites are located with Human *Leptin* complete CDS as reference and gaps are removed.
