# Supplementary material for: Adaptive Evolution of Leptin in Heterothermic Bats
Source: PLoS One. 2011 Nov 16;6(11):e27189. doi: 10.1371/journal.pone.0027189 (PMC3217946; doi:10.1371/journal.pone.0027189)
Supplement: Table S6 — Summary of amino acid variants specifically in heterothermic bats identified by the MAPP analysis. (DOC) [file pone.0027189.s010.doc]

**Table S6. Summary of amino acid variants specifically in heterothermic bats identified by the MAPP analysis.**

| Amino acid substitution | MAPP scores | Location and known function |
| --- | --- | --- |
| Q6 (L/Y) | 22.76/15.83 | AB loop, which is thought to help stabilize the conformation of Leptin. |
| L11 (F) | 12.59 |
| I14 (L/V) | 9.93/9.27 |
| L17 (F) | 8.31 |
| H18 (Y) | 12.96 |
| P19 (S) | 7.58 |
| L23 (M) | 9.73 |
|  |  |  |
| Q28 (D/E) | 34.35/26.89 | Helix B, which binds with OB-R and is involved in the metabolic regulation. |
| L30 (P) | 36.32 |
| A31 (E) ^c^ | 36.58 |
| V32 (T) ^c^ | 14.68 |
| Q35 (K) ^c^ | 16.54 |
|  |  |  |
| V45 (A) | 10.3 | Helix C, which binds with OB-R and is involved in the metabolic regulation. |
| I46 (M) | 8.59 |
| I48 (V) | 9.95 |
| L52 (M) | 10.65 |
| E53 (K) | 9.68 |
| D57 (E/H) ^a b^ | 23.98/20.32 |
| H60 (Q) ^a^ | 14.29 |
| K65 (N) ^a b c^ | 8.34 |
|  |  |  |
| P70 (H) ^a^ | 11.71 | CD loop, which regulate the energy metabolism by controlling food intake. |
| S73 (E/N/P) ^a^ | 9.65/16.41/33.15 |
|  |  |  |
| L83 (Q) ^a^ | 15.01 | Helix E, like CD loop can regulate the energy metabolism by controlling food intake. |
|  |  |  |
| S94 (D) ^c^ | 10.17 | Helix D. Might help bind with a receptor and stabilize protein conformation. |
| R95 (G) | 25.9 |
| D102 (T) | 9.1 |
| W105 (L) | 9.52 |
| D108 (I) | 23.98 |
| L109 (V/Y) | 10.52/ 14.95 |
| Mean±SE | 21.9±0.23 |  |

NOTE—All gaps are removed and amino acid numbers are based on the human orthologue of *Leptin* exon 3. The sites are localized to the functional domains based on the previous studies (Lee et al. 1996; Grasso et al. 1997; Zhang et al. 1997; Grasso et al. 1999; Hiroike et al. 2000). a: sites locate in the functionally significant fregamen 85-119; b: sites are binding with the receptor; c: sites are identical with the pika study (Yang et al. 2008).
